# Solar Blue Light Radiation Enhancement during Mid to Low Solar Elevation Periods under Cloud Affected Skies

**DOI:** 10.3390/s20154105

**Published:** 2020-07-23

**Authors:** Alfio V. Parisi, Damien P. Igoe, Abdurazaq Amar, Nathan J. Downs

**Affiliations:** 1 Faculty of Health, Engineering and Sciences, University of Southern Queensland, Toowoomba 4350, Australia; damienpaul@gmail.com (D.P.I.); abdurazaq.amar@usq.edu.au (A.A.); nathan.downs@usq.edu.au (N.J.D.); 2 Centre for Applied Climate Sciences, University of Southern Queensland, Toowoomba 4350, Australia

**Keywords:** cloud modification factor, visible radiation, instrumentation, high energy visible, blue light

## Abstract

Solar blue-violet wavelengths (380−455 nm) are at the high energy end of the visible spectrum; referred to as “high energy visible” (*HEV*). Both chronic and acute exposure to these wavelengths has been often highlighted as a cause for concern with respect to ocular health. The sun is the source of *HEV* which reaches the Earth’s surface either directly or after scattering by the atmosphere and clouds. This research has investigated the effect of clouds on *HEV* for low solar elevation (solar zenith angles between 60° and 80°), simulating time periods when the opportunity for ocular exposure in global populations with office jobs is high during the early morning and late afternoon. The enhancement of “bluing” of the sky due to the influence of clouds was found to increase significantly with the amount of cloud. A method is presented for calculating *HEV* irradiance at sub-tropical latitudes from the more commonly measured global solar radiation (300–3000 nm) for all cases when clouds do and do not obscure the sun. The method; when applied to global solar radiation data correlates well with measured *HEV* within the solar zenith angle range 60° and 80° (R^2^ = 0.82; mean bias error (MBE) = −1.62%, mean absolute bias error (MABE) = 10.3% and root mean square error (RMSE) = 14.6%). The technique can be used to develop repeatable *HEV* hazard evaluations for human ocular health applications

## 1. Introduction

The blue region (380−500 nm) of the visible waveband possesses the highest energy within the scotopic limits of human vision. The shorter blue-violet wavelengths within this range (380−455 nm) are sometimes referred to as high energy visible (*HEV*) radiation [1,2]. At the terrestrial surface, the action spectrum for blue light hazard peaks at approximately 440 nm [3,4] and is associated with what is frequently referred as the “blue light hazard”. In contrast to blue-violet wavelengths that have higher energy, the green-yellow wavelengths associated with photopic vision have lower energy as the photopic action spectrum peaks at about 555 nm. These wavelengths are beneficial to the regulation of human circadian rhythms and colour perception [2]. Recently, the International Commission of Illumination [5] issued a position statement to mitigate confusion and misuse of the term “blue light hazard”, stating that the term is only to be used in circumstances where actual retinal photochemical injury occurs. As this research does not measure retinal injury, the term “*HEV*” will be used throughout.

*HEV* transmits through the cornea and crystalline lens to the retina [1,2,3,6,7,8,9]. Intense or long duration of exposure to strong *HEV* sources is hazardous and could lead to photochemical damage of the retina. This damage is referred to as “photoretinitis” [4,7,10,11,12,13], also known as “photoretinopathy” [3,11] or photomaculopathy [5,14,15]. Prolonged exposure to *HEV* has the potential to contribute to the development of age related macular degeneration (AMD) [1,2,4,8,12,15], although this link has not been confirmed and is thus not universally agreed upon [5,8]. 

Children, the elderly and those with aphakic or pseudoaphakic eyes (those missing the crystalline ocular lens) are considered much more at risk of retinal harm [3,5,7,9,15,16,17]. There is evidence that vulnerability to overexposure to *HEV* may vary depending on individual dietary deficiencies [8]. Geographical altitude may also affect *HEV* exposure. Aviators are often subjected to enhanced exposures while at cruising altitudes [18,19]. However, there remains no conclusive evidence of any adverse permanent health effects of the exposure to blue light when it does not exceed exposure limits [5], which is stated by the International Commission on Non-Ionizing Radiation Protection (ICNIRP) as being 100 Wm^−2^sr^−1^ for long time viewing (greater than 10,000 s or approximately 2.8 hr) [3,17].

Many commonly used modern day devices, such as smartphones, tablets, laptops, LED lighting and vehicle headlamps have been identified as sources of *HEV*. These sources are often associated with the ‘blue light hazard’ following prolonged exposure [9,20]. Depending on the exposure period, the ocular dose due to exposure from artificial sources is often less than exposure due to natural sources, including blue sky light [2,3,5,17]. 

The blue appearance of the sky has long been explained by Rayleigh Scattering that correlates the intensity of scattered light (due to particles of size much smaller than the incident wavelength) with the inverse of the fourth power of the incident wavelength [21,22,23,24]. As the solar zenith angle (*SZA*) increases, the proportion of blue light (*HEV*) in the direct beam decreases relative to green and red, resulting in the predominantly reddish sunsets and sunrises [21,22]. The *HEV* irradiance available at the earth’s surface is affected by high and low altitude atmospheric factors, such as aerosol and particulate concentration and cloud amount and types [11,13,21,22,23,24]. The often visually bright “whiteness” observed in many atmospheric phenomena, such as clouds and fog, is due to relatively larger particles, such as water droplets, that enhance scattering across all solar wavelengths, described by Mie Scattering theory as being the result of a stronger forward scattering of incident radiation [21,22,23,25], thus increasing the amount of diffuse *HEV*.

Ocular exposure to *HEV* from direct observation of the sun of even less than a second can cause considerable harm [9,11,26]. However, this is not normal, and certainly is not a sensible behaviour. The human response is often to “squint”, shield the eyes or turn away [14]. By comparison, blue sky can be observed for extended timeframes, as cumulative *HEV* exposure is well below exposure limits [7,9,17]. There are several atmospheric constituents that absorb, scatter and attenuate visible wavebands, including nitrogen dioxide [27], and aerosols such as soot (“black carbon”) and desert dust [28,29,30,31,32]. The scattering ability of aerosols generally increases as wavelength decreases in the visible wavebands, enhancing the natural diffuse shortwave radiation [32].

Cloud variability and high *SZA* (above 70°) has been observed to cause an increase in uncertainty in solar irradiance observations, which includes *HEV* [13,33]. Some clouds, particularly thin clouds, can appear very bright when they are close to the vicinity of the sun [34,35,36], resulting in the forward scattering of sunlight through the optically thin clouds [34]. Similar visible solar radiation enhancements have been attributed to the reflection from the edges of sharply defined thicker, opaque clouds [18,34,35,37]. Short durations of increased solar irradiance are often observed when broken clouds intermittently pass by the sun. These durations can last from a few seconds to several minutes [34,36,37]. The intermittent increases in brightness can result in temporary visual disabilities and irritation [3].

The effects of cloud cover are typically measured for wavelengths shorter than the *HEV* in the ultraviolet radiation (UV) waveband. Cloud enhancement above cloud-free levels or attenuations in UV irradiance has previously been reported [38,39,40,41,42,43,44]. Cloud enhancement and attenuation of solar radiation, expressed with respect to a cloud-free sky, are often quantified by applying a cloud modification factor (*CMF*) [39,40,41,42,43,44,45,46]. 

The cloud-free reference irradiance is often modelled for the specific aerosol conditions expected at the date and time of observation [47]. The *CMF* is often less than one but can be greater than one if the irradiance at the time is enhanced due to the presence of clouds [48]. Solar ultraviolet radiation *CMF* is largely calculated by empirical determination using parameterisation, particularly using a least squares fit [49,50]. 

Cloud observations and *CMF* statistical evaluations have been performed in research to determine the relationships between *CMF* and the ultraviolet index [45], eye damage [50] and vitamin D effective UV irradiances [51]. No previous research has considered the influence of cloud on the *HEV* and this research extends *CMF* empirical determination into the *HEV* to derive a methodology for evaluating the *HEV* irradiances to a horizontal plane at sub-tropical latitudes under cloudy conditions at high *SZA* from 60° to 80°. The *HEV* irradiances measured during these periods are representative of adventitious ocular exposures that may be received when populations are in transit to and from work or school. They represent exposures received outside peak midday periods in tropical locations but are also representative of solar elevations experienced globally on a daily basis with most latitude ranges. For a working population, these exposures are typical of the ocular exposures an individual may receive over a lifetime through repeated travelling to and from work. The approach to be employed to evaluate the *HEV* irradiance to a horizontal plane under cloudy conditions is to develop a model with the *CMF* ratios of the global irradiance (300−3000 nm) and the *HEV* irradiance, taken as the effective irradiance weighted to the American Conference of Governmental Industrial Hygienists (ACGIH) spectral weighting function [11].

## 2. Materials and Methods

### 2.1. Instrumentation

All measurements and observations were made using an irradiance data acquisition system installed on a building roof at the University of Southern Queensland campus in Toowoomba, Queensland, Australia (27.56 °S, 151.95 °E, elevation 693 m). Toowoomba is an inland regional city at a sub-tropical Southern Hemisphere location with a population of the order of 169,000. The site is at an elevation of 693 m above sea level and has low atmospheric pollution levels. The data acquisition system consists of a Blue Light Safety Sensor (model PMA1121, Solar Light Co. PA. USA), Global Irradiance Sensor (model CMP3 Kipp and Zonen Pyranometer, Campbell Scientific Australia Pty Ltd., Garbutt, Australia) and Data Logger (model CR1000, Campbell Scientific Australia Pty Ltd., Garbutt, Australia). The instruments use the calibration provided by the manufacturer upon purchase. The blue light sensor measures the effective irradiance weighted by the American Conference of Governmental Industrial Hygienists (ACGIH) spectral weighting function for blue light hazard [52], while the CMP3 pyranometer measures the absolute global solar irradiance in the wavelength range 300 to 3000 nm. The two sensors are located side by side on a roof of a building at the University. The output signals of both sensors are logged at a scan rate of 50 Hz by the CR1000 data logger that is integrated with the sensors. Data were collected from October 2015 to May 2019.

The concurrent fractional cloud cover at the measurement site is quantified by a Total Sky Imager (TSI) (model TSI440, Yankee Environmental Systems, USA) installed in proximity to the PMA1121 Blue light safety sensor and CMP3 pyranometer. The TSI is programmed to provide the fraction of cloud cover by taking pictures of the sky and clouds reflected from a hemispherical dome and information on whether the solar disc is obscured by cloud [53]. Those images are sent to a workstation computer and processed to produce raw colour images that are analysed for fractional cloud cover. Pre and post processed images are stored as JPEG and PNG files, respectively, along with text files containing the extracted data.

The fractional cloud data with a value between 0 and 1 as it is provided by the TSI440 were also converted to okta classification according to the World Meteorological Organisation (WMO) standards [54] for consistency with the global standard. The cloud amounts in okta were employed to aggregate the data to allow consideration of the cloud amount on the influence on the *HEV* irradiance. The fractional cloud data which has the higher resolutions with decimal values between 0 and 1 were used in the development of the model between the global irradiance and the *HEV* irradiance. The details of the groupings based on the WMO cloud fraction classifications are shown in Table 1; it should be noted that each okta does not necessarily represent an eighth of the sky covered by clouds.

### 2.2. Data Analysis

The *SZA* range (60° to 80°) coincided with local times of before 9:00 a.m. and after 3:00 p.m. *HEV* data, global irradiance data and corresponding TSI440 image data were collected at five-minute intervals associated with the *SZA* interval. *HEV* data were recorded as a single text file. A script was developed to collect and collate the global irradiance and the *HEV* irradiance with the time stamped cloud fraction and sun visibility data from the TSI440 data files [55]. The method used to evaluate the *HEV* irradiance extends a model developed for the UV waveband to use a comparison between the global and *HEV CMF* ratios [41,42,46,49,50,51,56]. 

### 2.3. CMF Calculations

Data from the PMA1121 and CMP3 recorded from October 2015 to the end of June 2018 were used to develop a model for *HEV* irradiance. Data from the start of July 2018 to mid-May 2019 were used for validation. Adapted from prior research for UV radiation, the *HEV* irradiances (*E_HEV_*) were determined using a cloud modification factor (*CMF_HEV_*) as in Equation (1) [39],

(1) $$E_{HEV}=CMF_{HEV}\times E_{HEV}^{Sky}$$

where $E_{HEV}^{Sky}$ is the *HEV* irradiance for cloud-free skies. There are two main methods that have been used in previous solar UV research to determine cloud effects and cloud-free conditions—the first is the calculation of a clearness index, based on a comparison with extra-terrestrial irradiance at the target wavelengths [41,46,55]; the second bases cloud-free sky determination on TSI cloud fraction data [42,45,50,51]. Given the availability of corresponding TSI cloud fraction data, $E_{HEV}^{Sky}$ data were determined when the cloud fraction was less than 0.02 or approximately 0 okta [51]. 

A function for $E_{HEV}^{Sky}$ was developed using a power law model used for solar irradiance compared with the cosine of the respective *SZA* [49,57], and represented by converting the *SZA* to an effective air mass (*m*) in Equation (2) [58]:

(2) $$m=\frac{1}{\cos\left(SZA\right)+0.50572{\left(96.07995-SZA\right)}^{-1.6364}}$$

For the *SZA* range of 60° to 80°, the second term in the denominator of Equation (2) is smaller than 0.006. Equation (2) was, therefore, approximated in this research by:

(3) m=sec(SZA)

**Figure 1:**
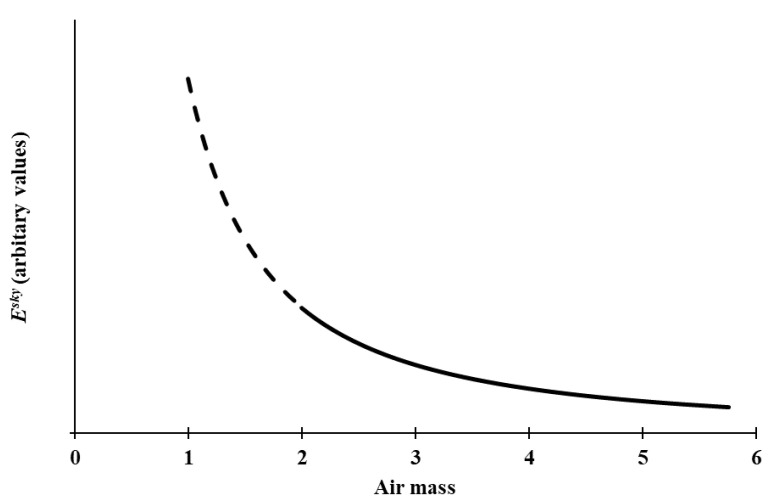
Generalised effect of the air mass on the derived clear sky irradiance (*E^sky^*). The solid line represents the expected values for the air mass in this research.

Thus, the power law relationship for the cloud-free sky *HEV* may be expressed according to Equation (4) [51].

(4) $$E_{HEV}^{Sky}=am^{b}$$

where *a* and *b* are curve fitting parameters. In previous research in the UV waveband [50,51], *b* is typically a negative value. The shape of the derived relationship is shown in Figure 1. This shape is generally the same as that observed in previous research [41,46,51,56], demonstrating a sharp increase in the derived clear sky irradiance as air mass decreases towards zenith, particularly for air masses less than 2 (*SZA* < 60°). Comparatively, a much more subtle increase is apparent in the air masses observed in this research (as indicated by the solid line in Figure 1).

In previous research observing UV *CMF*, two curve fitting methods were employed to develop models based on an exponential [41] and a power law [46] relationship between UV and Global *CMF* (*CMF_G_*). These methods have been modified for modelling *HEV CMF* (*CMF_HEV_*) in Equations (5) and (6).

(5) $$CMF_{HEV}=\alpha \left(1-e^{\beta \left(CMF_{G}\right)}\right)$$

(6) $$CMF_{HEV}=\gamma {\left(CMF_{G}\right)}^{\delta }$$

where *α*, *β*, *γ* and *δ* are curve fitting parameters.

The cloud modification function for global broadband solar radiation (*CMF_G_*) was determined as a ratio between the measured global solar irradiance in all cloud conditions (*G*) and that in a cloud-free sky (*G^Sky^*) [41,50,51], taken as a cloud fraction of less than 0.02 [51], in Equation (7).

(7) $$CMF_{G}=\frac{G}{G^{Sky}}$$

The cloud-free global irradiance (*G^Sky^*) was parametised by Long and Ackerman [57] and Pfister et al. [49] and adapted in Equation (8):

(8) $$G^{Sky}=c{\left[\cos\left(SZA\right)\right]}^{d}$$

where *c* and *d* are curve fitting parameters. The data for equations 4 and 8 for the cloud-free sky cases were analysed to determine the constants *a*, *b*, *c* and *d*.

## 3. Results 

### 3.1. Cloud-Free Irradiance 

**Figure 2:**
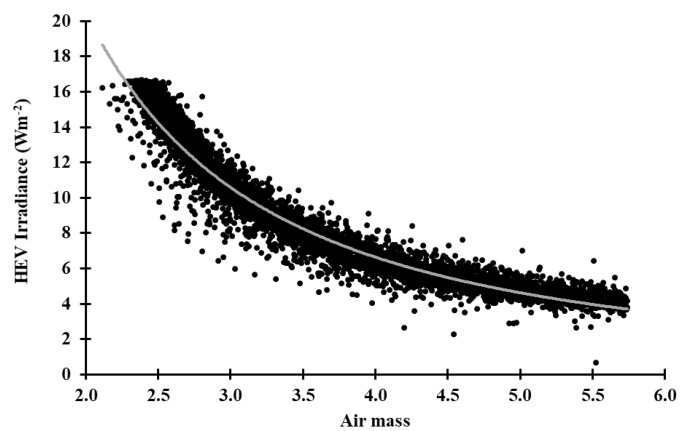
High energy visible (*HEV*) cloud-free sky irradiance modelled with respect to optical air mass (n = 5975). The grey line represents the fitted power law model.

Figure 2 presents the *HEV* cloud-free sky irradiance ($E_{HEV}^{Sky}$) plotted versus air mass in the range 2.0 to 5.8. The cloud-free cases (n = 5975) were taken for the times when the cloud fraction was less than 0.02.

The *HEV* cloud-free sky irradiance model (Equation (9)) provides a very strong fit to the data (R^2^ = 0.94) (Figure 2), consistent with the expected shape in Figure 1 and congruent with previous work modelling cloud-free solar irradiance data [51].

(9) $$E_{HEV}^{Sky}=62.70m^{-1.62}$$

The global irradiance cloud-free sky model fitted to the corresponding cloud-free global irradiance is represented in Equation (10), with an R^2^ = 0.85.

(10) $$G^{Sky}=260.18m^{-1.34}$$

### 3.2. Modelling HEV Data

**Figure 3:**
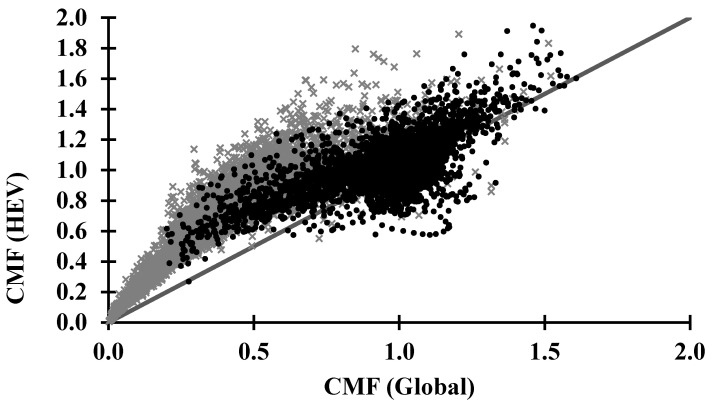
Comparison of the cloud modification factor (*CMF*) for *HEV* and for global irradiances in all conditions. Black dots represent data collected when the solar disc was visible (n = 8384) and grey crosses where it was obscured (n = 5780). The black line represents a 1 to 1 relationship between *HEV* and global *CMF* ratios.

A comparison between the *CMF* for *HEV* and for global irradiance under all conditions for data collected from October 2015 to June 2018 is presented in Figure 3. The data have been separated according to solar disc visibility [42].

Figure 3 shows the same pattern of the daily, seasonal and local variances associated with local cloud cover conditions—observed by previous research [41,42,46,51] for UV irradiance. The relationship exhibited in Figure 3 provides important information related to solar disc visibility. Two regions are apparent in Figure 3. The first area is an irregular accumulation of data centred at approximately *CMF* = 1 for both global and *HEV* irradiance observations corresponding to when the sun was visible in the sky, suggesting that the *CMF* is dominated by the direct solar radiation. However, for the second group of data when the solar disc is obscured, the clouds have a larger relative influence on the *HEV* radiation compared to the global radiation. 

**Figure 4:**
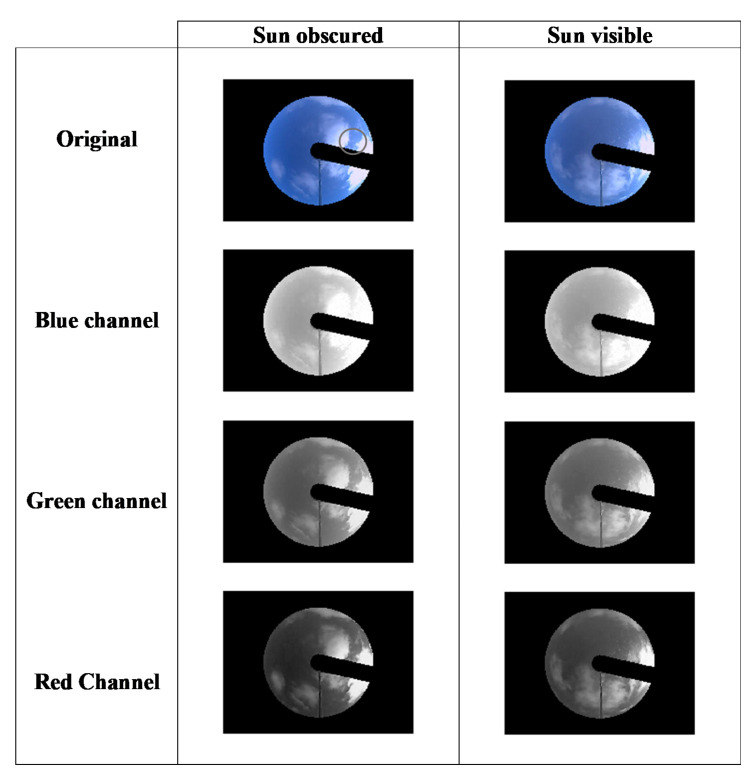
Example of consecutive (one-minute interval) high solar zenith angle (*SZA*) (62°) total sky imager (TSI) images. The blue sky is brighter in the blue channel for both the sun-obscured and the sun-visible cases due to the higher relative proportion of Rayleigh scattering at the shorter wavelengths.

The *CMF_HEV_* data are considerably greater than the *CMF_G_* observations. Further evidence of the increased scattering of *HEV* due to clouds is presented in consecutive (one-minute interval) TSI images where the sun progresses from being obscured to visible in Figure 4. The black band in the images represents the band on the hemispheric dome of the TSI440 that is designed to ensure that the image of the sun is blocked and not imaged by the camera. This band is on the hemispheric dome which does a full rotation once every 24 h. The thin black band is the shadow of the metal arm that holds the camera over the hemispheric dome. In the images, the visually cloud-free part of the sky in the vicinity of the sun in the sun-obscured and sun-visible images (Figure 4) demonstrate significant brightening predominantly in the blue channel for both the sun-visible and the sun-obscured cases, demonstrating an enhancement of the shorter wavelength diffuse Rayleigh Scattering. Neighbouring clouds are considerably brighter in the blue channel compared to the green and red.

**Figure 5:**
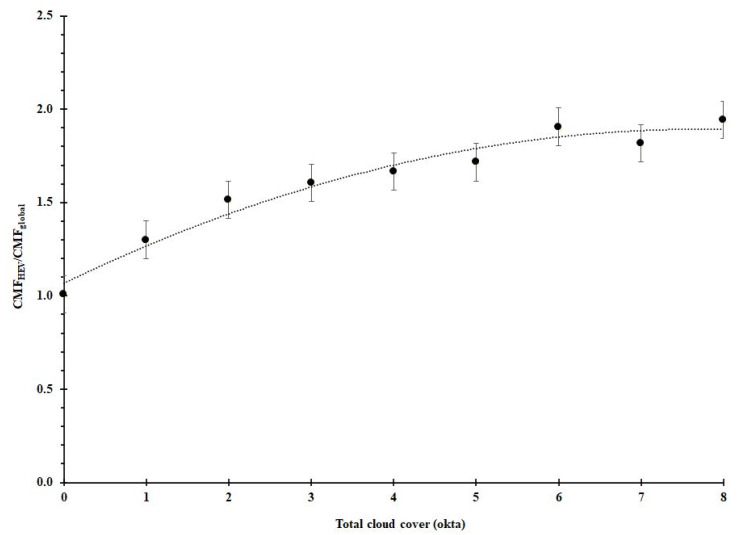
Comparison between the ratio of *HEV CMF* and global *CMF* with cloud fraction measured in okta for all data (n = 23,500).

A comparison between the *CMF* ratio ($\frac{CMF_{HEV}}{CMF_{G}}$) with measured cloud fraction, measured in oktas (Table 1), is presented in Figure 5. Each data point is the average of the data for the cloud fraction range for the relevant okta and the error bars represent one standard error of the data. Generally, it is very clear that in the presence of any amount of cloud, $CMF_{HEV}$ is consistently greater than $CMF_{G}$ indicating greater *HEV* irradiance enhancement due to the influence of cloud relative to the global irradiance.

The exponent model developed in the literature for UV irradiance (Equation (5)) was adapted to predict the *HEV CMF* ratios with respect to global conditions (Equation (11)). There exists a strong relationship, with R^2^ = 0.79.

(11) $$CMF_{HEV}=1.13\left(1-e^{-2.62(CMF_{G})}\right)$$

The power law model adapted from Equation (6) demonstrates a stronger relationship between *HEV* and global *CMF* ratios (Equation (12)) with R^2^ = 0.86. Incidentally, the power law model developed compares with that determined for UV observations [46,56].

(12) $$CMF_{HEV}=1.09{\left(CMF_{G}\right)}^{0.60}$$

Consequently, the power model was used for additional analysis; the *HEV* irradiance can, therefore, be calculated from global irradiance for all conditions (*G*) and optical air mass (*m*) between approximately 2.0 to 5.8 in a combined *HEV* irradiance model (Equations (13)–(15)) using the general *HEV* irradiance model (Equation (1)). Substituting the derived models for *HEV CMF* (*CMF_HEV_*—Equation (12)) and *HEV* cloud-free irradiance ($E_{HEV}^{Sky}$—Equation (9)) yields an intermediary expression for deriving the *HEV* irradiance using global *CMF* (*CMF_G_*) and air mass (*m*):

(13) $$E_{HEV}=1.09{\left(CMF_{G}\right)}^{0.60}\times 62.70m^{-1.62}$$

The model developed in this research through substitution for *CMF_G_* using Equations (3), (7), (10) gives:

(14) $$CMF_{G}=\frac{G}{260.18m^{-1.34}}$$

Allowing simplification to the model:

(15) $$E_{HEV}=2.43G^{0.60}m^{-0.82}$$

### 3.3. Validation

**Figure 6:**
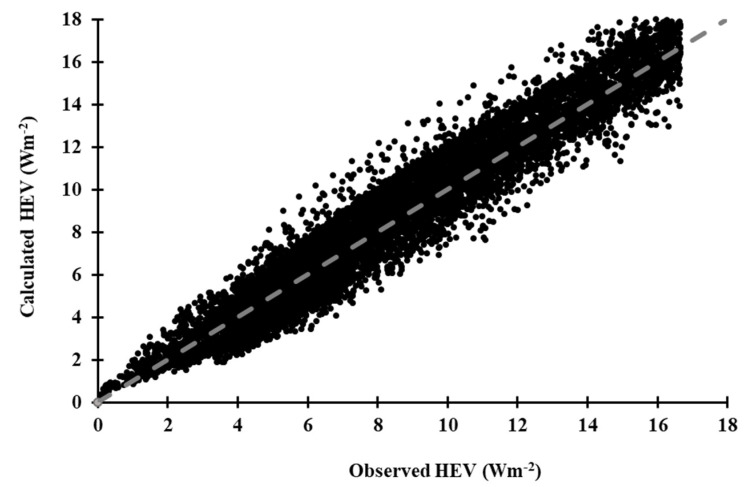
Comparison between observed and calculated *HEV* irradiance between July 2018 to May 2019 (n = 9336). The dashed line represents a one to one fit with the data.

*HEV* irradiance and the associated *CMF_HEV_* ratios from July 2018 to May 2019 were used to validate the modelled *HEV* irradiances in Equation (15) (Figure 6). The dashed line is the one to one line to enable the comparison between the modelled *HEV* irradiance and the measured *HEV* irradiance.

The calculated *HEV* irradiances are in very good agreement with the observed values (Figure 6) with an R^2^ = 0.82, mean bias error (MBE) of −1.62%, mean absolute bias error (MABE) of 10.3% and a root mean square error (RMSE) of 14.6%. This validation was found to be reasonably comparable in these statistical measures with previous equivalent models [46,56] for equating UV irradiance in all conditions, indicating a very high degree of robustness with the model, allowing its use for predicting *HEV* irradiance. A comparison between the statistical evaluations of the *HEV* and equivalent similar models [46,56] is presented in Table 2. Although the *HEV* is a different waveband to the UV, it is provided here as an indication of the expected MBE, MABE and RMSE with the approach employed in this research.

## 4. Discussion

The cloud-free *HEV* irradiances in Figure 2 have a small amount of scatter about the fitted curve. This is due to minor atmospheric variations, such as possibly thin high-level clouds that are not always detected by the TSI440 sky camera. There can also be variations in atmospheric aerosols that influence the *HEV* irradiance. Nevertheless, the fitted power law model with an R^2^ = 0.94 provides the basis for the calculation of clear sky irradiance as a function of the air mass.

The *CMF* for *HEV* and for global irradiances in Figure 3 show that when the solar disc is obscured, the *CMF_HEV_* observations are considerably greater than *CMF_G_* observations. Most of the data were above the one to one line, indicating enhancement of the *HEV* relative to the global irradiance. This is likely due to significant effects of reflection and the increased scattering of *HEV* due to clouds, particularly if the clouds are thin or the sun is near the edge of clouds.

Figure 5 shows that for the data sets at each okta there is a variation in the ratio of ($\frac{CMF_{HEV}}{CMF_{G}}$). A sensitivity analysis found that using the cloud fraction at each 5- or 10-min interval served as a strong indicator of the average cloud cover during the 5 min interval, especially when the fraction was categorised according to the WMO cloud cover classifications. The variation in the *CMF* ratio for each okta that is represented by the error bars is due to the variations in cloud properties such as cloud thickness, cloud height, cloud proximity to the sun, cloud orientation with respect to the sun and whether or not the solar disc is obscured. However, there is a definite upward trend in the ratio of the averages at each okta. This is essentially cloud enhancement of the *HEV* irradiance that increases with increasing cloud fraction. The only time when the *CMF* ratio is at unity ($\frac{CMF_{HEV}}{CMF_{G}}\approx 1$) is in completely cloud-free skies (0 okta). Even for cloud levels as low as one okta, the $\frac{CMF_{HEV}}{CMF_{G}}$ average is higher than 1.2. This increases to approximately 1.94 for 8 okta of clouds. 

The cloud enhancement in the *HEV* waveband, as shown by the average of the ratio of the cloud modification factor of the *HEV* waveband to the global radiation waveband for 8 okta of clouds of 1.94 is significantly higher than the cloud enhancement in the UV waveband that has been previously found with an enhancement above that of clear sky irradiances ranging up to a factor of 1.28 [53]. This results in any part of the population that are sensitive to *HEV* irradiance experiencing a potentially larger effect due to the *HEV* waveband on cloudy days.

The validation of the model developed in this research for low solar elevation angles (solar zenith angles between 60° and 80°) during the early morning and late afternoon when a significant part of the population are going to work or taking children to school shows that the MBE values were very similar to those developed for the UV waveband. The *HEV* irradiance model was within the range of errors determined by Villán et al. [46] for the UV waveband. The elevated MABE and RMSE for the *HEV* model compared to previous research can be accounted for by the differences in methodology. Most notably the cloud data used in this research was obtained from a TSI. The model developed is relevant for sub-tropical latitudes and will need further investigation at other latitudes where there may be different cloud types and the *SZA* will be different for when the population are going to work or taking children to school. Additionally, the influence of cloud proportion with respect to the position of the sun can also be investigated in future research.

## 5. Conclusions

A model was developed and validated for the evaluation of the solar *HEV* to a horizontal plane at a sub-tropical latitude for *SZA* between 60° and 80° for all cloud conditions. The method which incorporated both the effect of clouds on global solar radiation and the air mass was developed using approximately 26 months of 5-min interval data at high *SZA*. The validity of the model was tested using a new data set of approximately 11 months, providing an R^2^ = 0.82, a mean bias error (MBE) of −1.62%, mean absolute bias error (MABE) of 10.3%, and a root mean square error (RMSE) of 14.6%.

Clouds were found to form a significant influence on the *HEV* with a greater influence of clouds on the blue light compared to their influence on global radiation, increasing significantly when there were increased amounts of clouds and also when the solar disc was obscured by clouds. In some climates, especially in tropical climates, there is more cloud cover than in temperate zones increasing the likelihood that cloud enhanced glare would be experienced. Therefore, based on our preliminary findings the use of *HEV* blocking glasses is likely to remain recommended for ocular health and comfort.

The method developed in this research provides a means for calculating *HEV* to a horizontal plane utilising data obtained from the global solar radiation and the air mass. Although, this is not direct exposure to the eyes as the eyes are predominantly oriented below the horizontal plane, the *HEV* received on a horizontal plane is a logical first step in determining *HEV* exposures to the eyes. Data on the global solar radiation are typically collected at more sites than the *HEV*, with very few sites collecting data on the *HEV*; thus, this research presents a model that calculates the *HEV* from global radiation data at sites where *HEV* data are not recorded. Any available global radiation data can, therefore, be used to reconstruct the *HEV* to within a reasonable level of certainty, enabling continued research on the effects of HEV on human health.

## Figures and Tables

**Table 1:** Cloud fraction classification to okta used in this research to correspond with the World Meteorological Organisation classifications.

Okta	WMO Cloud Fraction	This Research Cloud Fraction
0	0	<0.02
1	Up to 1/10, but not 0	0.02 to < 0.15
2	2/10–3/10	0.15 to < 0.35
3	4/10	0.35 to < 0.45
4	5/10	0.45 to < 0.55
5	6/10	0.55 to < 0.65
6	7/10–8/10	0.65 to < 0.85
7	Greater than 9/10 but not 10/10	0.85 to 0.98
8	10/10	>0.98

**Table 2:** Statistical evaluation comparison of the *HEV* irradiance (*HEV*) model with research in the UV waveband.

	*HEV* Irradiance Model	UV Model 1 Villán et al. [46]	UV Model 2 Wang et al. [56]
**MBE**	−1.62%	−2.71% to 1.86%	−0.91% to 1.52%
**MABE**	10.3%	6.77% to 7.82%	8.76% to 9.18%
**RMSE**	14.6%	9.83% to 10.46%	11.13% to 12.32%
